# Redistributive effects of health care out-of-pocket payments in Cameroon

**DOI:** 10.1186/s12939-021-01562-8

**Published:** 2021-10-18

**Authors:** Augustin Ntembe, Regina Tawah, Elkanah Faux

**Affiliations:** Bowie State University, Bowie, MD USA

**Keywords:** Equity, Health care financing, Out-of-pocket payments, Redistributive effects, Income inequality

## Abstract

**Background:**

The bulk of health care financing in Cameroon is derived from out-of-pocket payments. Given that poverty is pervasive, with a third of the population living below the poverty line, health care financing from out-of-pocket payments is likely to have redistributive and equity effects. In addition, out-of-pocket payments on health care can limit the ability of households to afford non-healthcare goods and services.

**Method:**

The study estimates the Kakwani index for analyzing tax progressivity and applies the model developed by Aronson, Johnson, and Lambert (1994) to measure the redistributive effects of health care financing using data from the 2014 Cameroon Household Survey. The estimated indexes measure the extent of the progressivity of health care payments and the reranking that results from the payments.

**Results:**

The results indicate that out-of-pocket payments for health care in Cameroon in 2014 represented a significant share of household prepayment income. The results also show some evidence of inequity as few people change ranks after payment despite the slight progressivity of health care out-of-pocket payments.

**Conclusion:**

The existence of some disparities among income groups implies that the burdens of ill-health and out-of-pocket payments are unequal. The detected disparities within income groups can be reduced by targeting low-income groups through increases in government expenditures on health care and pro-poor prioritization of the expenditures.

## Introduction

Although the public sector is the most important provider of health services in Cameroon, an estimated 69.63% percent of the funding for the health sector was generated from out-of-pocket payments in 2018. The problem of poverty is pervasive, and about 37.5% of the population lives below the poverty line, according to the Cameroon Household Survey conducted in 2014. Health care financing with the participation of the population is likely to have redistributive effects and equity consequences. Out-of-pocket payments on health care reduce the amount of disposable income available to households or individuals after health care payments, thus limiting the use of health care services and widening the gap between the poor and the rich. Analogous to taxes, out-of-pocket expenses on health care affect the household proportionately, progressively, or regressively depending on the structure of the health payment system.

The study, unlike others, examines the extent of inequality in the distribution of incomes arising from the prevailing health care financing arrangements in Cameroon. In particular, the study investigates whether or not health care financing in the country through out-of-pocket household payments bridge or widens the gap between poor and rich households. A common way to achieve this objective is to compare the post-payment income distribution with prepayment distribution to gauge the extent of inequity created by out-of-pocket payments. Furthermore, the study determines the extent of horizontal inequity or inequity within bands of equal incomes and the reranking of individuals according to post-payment incomes.

Based on data drawn from the 2014 Cameroon Household Survey, the study uses an index developed by Kakwani [12] for analyzing tax progressivity and the model developed by Aronson, Johnson, and Lambert [1] to measure the redistributive effect of out-of-pocket payments for health care. By investigating whether or not health care out-of-pocket payments bridge or widen the gap between poor and affluent households, the study attempts to fill the knowledge gap in the equity effects of health care out-of-pocket payments in Cameroon.

The rest of the paper is organized as follows. Section 2 presents a short description of the health care system in Cameroon. Section 3 presents the theoretical and empirical literature on equity in health care financing. Section 4 describes the methodology outlining the approaches used in measuring the progressivity of health care payments and the redistributive effects of such payments. Section 5 provides the empirical results, and a discussion of results follows in section five. Finally, the paper ends with a conclusion in section 7.

### Cameroon health care system

The Cameroon health care system is structured into the central, intermediate, and peripheral levels. The system is further subdivided into a public sub-sector, a private sub-sector, and a traditional sub-sector under the Ministry of Public Health (MOH [18]). Each of the three levels of the Cameroon health care system has administrative, health, and dialogue structures.

The central level is at the top of the Cameroon health system and includes the central services of the Ministry of Public Health. The central level coordinates, regulate and develop the country’s health sector strategies and policies. Some of the key structures that provide care at the central level include the General Reference Hospitals, the University Hospital, the Central Hospital, and agencies under the purview of the Ministry of Public Health such as the Essential Drugs Procurement Center, Centre Pasteur du Cameroun, the Gynecological Endoscopic Surgery and Human Reproductive Teaching Hospital among others. The dialogue structures consist of the National Council of Health Hygiene and Social Affairs.

The administrative structures at the intermediate level consist of the ten regional delegations of public health. The regional delegations of public health provide technical support to health districts. Health care is provided at this level by regional hospitals and assimilated structures. The Regional Fund for health promotion is an important health and dialogue structure.

The district level is represented by the health district services, and their role is to provide health care, coordinate and implement national health programs. Health services are provided at this level by district hospitals, medical centers, and district health centers. In 2016, there were a total of 189 health districts in Cameroon. Dialogue structures at the peripheral level of health include district health committees, district management committees, local health area committees, and district hospital management boards.

### Health care financing from out-of-pocket payments

Out-of-pocket payments also include direct payments to not-for-profit providers such as mission facilities and other for-profit health care providers ranging from services provided in public facilities, doctors working in private practice to informal drug vendors and traditional healers. These payments are made at the points of service, and the amount paid for medical care depends on the severity of illness, the point of service, and the patient’s ability and willingness to pay. The ability to pay for health services depends on income.

**Fig. 1:**
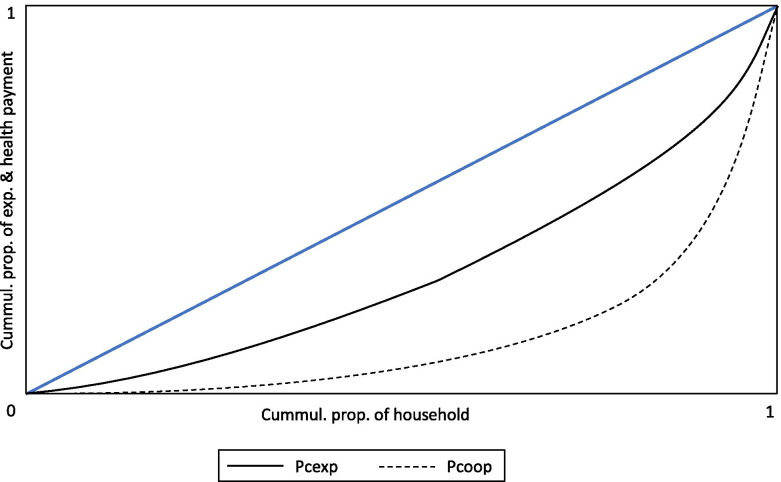
Lorenz and health payments concentration curves, Cameroon 2014. Source: Author’s computation based on data from the 4th Cameroon Household Survey, 2014

Table 1 shows that households are by far the most important source of health care expenditure in Cameroon. The level of household expenditures is reflected in the value of private expenditure as a percentage of total health care expenditure. The total household expenditures were estimated at 70.2% of the total healthcare expenditures in 2010 and 69.6% in 2018. (Fig. 1). The increase in health care cost finance from out-of-pocket payments, and the growing cost of health services in Cameroon are likely to affect households, especially those living below the poverty threshold, adversely. Also, the high out-of-pocket payments and declining government expenditures on health care increase financial barriers and reduce affordability and access to health services, especially for the poor. Insufficient and low public expenditure allocations on health care is one of the main health care financing problems plaguing health care systems in Africa [3] (Table 2).

**Table 1:** Organization of the Cameroon Health System

	Structures		Sub-sectors	Functions
Level	Administrative	Health Care		
Central	Minister’s Office, Secretariat General, Department, and Similar Structures	General hospitals; Central hospitals, and other structures ranked as such, for example, National Essential Drug Procurement Centre	Public, private, Traditional	Development of concept, policies and strategies, coordination, Regulation
Intermediate	10 Regional Delegations	Regional hospitals and others ranking as such, for example, Regional Drugs Supply Centers	Public, Private, Traditional	Technical support to health districts
Peripheral	189 Health Districts	District hospitals, sub-divisional medical centers, integrated health centers,	Public, private, Traditional	Implementation of programs

Source: Ministry of Public Health, HSS 2016–2027

**Table 2:** Health Care Financing Sources (in millions of constant 2018 US$

Year	External	Government	Health insurance	NGOs	Companies	Out-of-pocket	Total
2010	82.96	172.19	10.89	30.85	26.62	763.82	1087.35
2011	135.88	190.76	9.01	111.58	27.72	778.53	1253.47
2012	93.11	192.91	74.84	96.07	28.98	791.95	1277.86
2013	84.84	201.17	79.27	40.20	30.70	809.10	1245.28
2014	135.78	271.30	87.13	66.67	32.50	824.81	1418.19
2015	127.32	200.13	92.57	60.59	34.34	898.14	1413.08
2016	131.39	209.51	96.91	61.51	35.94	934.76	1470.01
2017	123.67	126.24	95.16	55.31	37.21	984.09	1421.68
2018	116.69	151.60	92.63	50.44	38.72	1031.80	1481.88

World Bank Atlas Sources – Knoema, 2021

Despite the rising burden of out-of-pocket payments, household participation in the financing of the health care system can generate additional resources for the public health sector and could potentially increase the utilization and the quality of health services (Litvack and Bordart [13]; Ntembe [22]). However, low-income households disproportionately face the brunt of the rising cost of health care through out-of-pocket payments with escalating impoverishing effects [10, 16, 19]. The burden of payments can be alleviated through the implementation of prepayment schemes such as social insurance that can provide funding support to health care [16]. However, social insurance schemes in Cameroon are less developed and only cover some workers in the formal system.

### Review of previous literature

The literature that examines equity in health care financing is quite recent. Developed from public finance literature, it analyses the extent to which the tax system redistributes income and wealth ([1, 12]; Wagstaff 1998; Wagstaff and Doorslaer [20, 25, 29]; Ataguba et al. [4]). Studies on the progressivity of the tax system have been extensive in developed countries and have relied on the mathematical model developed by Kakwani [12] for analyzing tax progressivity. Aronson et al. [1] have also developed a model to decompose the redistributive effects of taxation.

The models developed by Kakwani and by Aronson and others have been extended to the field of health care financing (Wagstaff et al. [33,24, 30]; Ataguba et al. [4]). The distribution of health care payments among households determines how their overall welfare is affected when they pay for health care. Therefore, equity concerns regarding the burden of health care payments are critical when deciding on health care financing options. Equity in health care financing is the extent to which the various health care payment options contribute to the redistribution of income (Deaton and Muellbauer [7]).

Health care payments can be progressive or regressive depending on whether the burden falls on richer or on low-income individuals. While progressive and regressive payments have opposite effects on the distribution of incomes, progressive payments reduce post-payment income inequality, whereas regressive payments increase post-payment inequality ([1, 28]; Ataguba & Akazili, 2010). Wagstaff et al.[33] used the Kakwani index of progressivity to find that total contribution to health care financing in the United States of America and the Netherlands were regressive, and the regressivity was more severe in the United States (− 0.15) than in the Netherlands (− 0.06). However, health care payments were found to be progressive in the United Kingdom (0.03).

In a study of the progressivity of health care financing mechanism, catastrophic spending on health, and the distribution of healthcare benefits in Ghana, South Africa, and Tanzania, Mills et al. [17] found that the overall healthcare financing was progressive in all three countries. The findings also indicated that out-of-pocket payments, in particular, were regressive in all three countries. In addition, the overall distribution of health service benefits in all three countries benefited the rich more than the poor, although the burden of illness was greater for lower-income groups.

Earlier studies found that out-of-pocket payments were progressive in Sierra Leone (Fabricant et al. [8]) and Burkina Faso (Makinen et al. [14]). In Mexico and Thailand, the poor were also found to be spending a higher proportion of their income on out-of-pocket payments than the rich [9, 23]. Further evidence from a World Bank study of the redistributive effects of health care payments in Vietnam using the Aronson decomposition revealed that health care payments adversely affected income distribution [26, 32]. In a recent study, Munye and Briggs (2014) analyzed the progressivity of the main sources of health care financing in Kenya. The authors used data from the Kenyan National Accounts of 2005–2006 and the Kenyan household expenditure and utilization survey conducted in 2007 to show that the overall Kenyan health care financing system is regressive. The study also showed that out-of-pocket payments on health care were regressive.

Ataguba et al.[4] used the Gini index to study the redistributive effects of health financing between and within groups in Nigeria. The results indicate that health care financing through out-of-pocket contributed to a significant increase in income inequality in Nigeria. The study also shows that, income inequality would be lesser within the six geopolitical zones in Nigeria without out-of-pocket payments. Thus, regardless of whether the health care financing system is progressive or not, out-of-pocket payments will lead to inequity in health care payments within the same income groups [19].

McIntyre et al. [15] have reported that out-of-pocket payments are the single largest source of health care financing in many African countries and impose a very heavy burden on households, particularly the poorest. 

Ataguba and McIntyre [5] used nationally representative datasets and standard methodology to examine equity in the delivery and financing of health care in both the public and the private sectors in South Africa. The study suggests an overall progressive financing system but a pro-rich distribution of health care benefits where more rich people than the poor benefit from the financing system. The study further suggests that the distribution of health care benefits is pro-rich but not according to health care needs. Richer groups receive a far greater share of service benefits within the public and private sectors, although with a relatively low burden of ill-health.

In most developing countries and especially in Africa, where prepaid financing of health care is limited, low-income households are likely to be disproportionately hurt by reforms that implement user charges for health services. Data from the Cameroon National Health accounts as well as from the Cameroon Household and Consumption survey published in 2014 suggest that over 70% of health care expenditure in Cameroon is financed from out-of-pocket household payments. Out-of-pocket payments are expected to be regressive, especially if lower-income groups are paying a large share of their incomes for health care.

## Methodology

The method adopted for this study is borrowed from the literature on tax equity, where income redistribution is associated with tax payments ([21]; Reynolds and Smolensky 1970 [1];). The literature extends to health care, where out-of-pocket payment is considered a payment that reduces the ability of the individual to purchase other goods and can lead to post-payment inequity. Health care payments can be regressive or progressive. The progressivity of health care payments is the extent to which health care payments rise or fall as a proportion of income as the latter rise or fall [30].

Thus, to investigate the impact of out-of-pocket household payments on equity in Cameroon, the study uses the Aronson decomposition to measure the redistributive effects of health care payments and the effects of payments on the distribution of income. The study measures the redistributive effect of the average proportions of incomes spent on health care, the progressivity or regressivity of the payment structure, the horizontal inequities in the financing system, and the extent of reranking generated from the payments. Re-ranking is the change in the order of income distribution that results from health service payments.

The health care payment system is progressive if health care payments rise by a higher proportion as income increases and regressive if the proportion of health care payment increases as income decreases. Health care payments can lead to a redistributive effect (*RE*). The RE of health care payments is simply the change in income inequality resulting from payments. The *RE* effect will be measured using the Lorenz and Gini coefficients by subtracting the post-payment Gini from the prepayment Gini coefficient.

### Kakwani progressivity index

Progressivity is often measured using an index proposed by Kakwani [12]. The Kakwani index measures the departure from proportionality as the difference between the concentration coefficients of payments and the Gini of prepayment income. It is calculated as,

1 $${\pi}_K={C}_T-{G}_X$$

where *C*_*T*_ is the health care payment concentration index, and *G*_*X*_ is the Gini of prepayment income. The value of π_K_ ranges from − 2 to 1 so that a negative number indicates that the payment is regressive, zero if proportional, and positive if progressive. The Kakwani index is derived from the principle of the Lorenz curve such that

2 $${\pi}_K= Lc(p)- Lx(p)$$

where *Lx(p)* is the Lorenz for prepayment income, and *Lc(p)* is the concentration curve for health care payments. The health care system is proportional when *Lx(p)* and *Lc(p)* are equal and equal to zero. Therefore, a departure of *Lc(p)* from *Lx(p)* is a measure of progressivity.

### Decomposing the redistributive effect of health care payments

Households’ payments for health care secure access to health services and may also redistribute incomes. The extent to which income redistribution occurs has important implications for the distribution of goods and services other than health care (Wagstaff [35]; Wagstaff and Doorslaer [27]). Although health care payments in some African countries have been largely progressive and pro-poor with more benefits accruing to the poor, the burden of illness has been greater for lower-income groups who face disproportionately limited access to health care services [2, 17, 19]. The impediments to expanding access to health care for the poor must be dismantled to allow universal coverage [2].

### The Aronson-Johnson-Lambert decomposition

The redistributive effect (*RE*) of health care payments can be measured by comparing the Gini coefficient of prepayment incomes with that from post-payment incomes as follows:

3 $$RE={G}^x-{G}^{x-p}$$

where *G*^*x*^ and *G*^*x-p*^ are the prepayment and post-payment Gini coefficients, x stands for prepayment income, and *p* denotes the payment. Following Aronson et al. [1], *RE* is simply the difference between the Gini coefficient for prepayment income and the Gini coefficient for post-payment income and is equal to the following:

4 $$RE=V-H-R$$

where, the vertical income redistribution $V=\left(\frac{g}{1-g}\right){\pi}_K$ represents the change in income inequality that results from health care payments if everyone at each prepayment income level had paid the same amount towards health care, g is the average share of prepayment income absorbed by health care payments. *H* is the effect of horizontal inequity, and *R* is the degree of re-ranking of households compared to the distribution before paying for health care. *R* will be zero if no such re-ranking occurs. A redistributive effect that is greater than zero implies that inequity in post-payment income is lower than in prepayment income. The pro-poor redistribution implies that post-payment income redistribution is in favor of the poor. Aronson et al. [1] express the *RE* in full as follows:

5 $$RE=\left(\frac{g}{1-g}\right){\pi}_t^k-\sum {\alpha}_x{G}_{F(x)}-{G}_{x-T}-{C}_{x-T}$$

The first term on the right of eq. (4) estimates the level of inequality that would result if everyone in each income band makes equal payments to the health care financing system. The term *G*_*F*(*x*)_ is the Gini coefficient that measures inequality in the post-payment period that arises when individuals with the same prepayment income level are now less equal because the individuals are contributing unequally to finance the health care system. The horizontal inequity (*H*) in each income band is measured by the weighted sum of the groups (j) specific post-payment Gini coefficients, ${G}_j^{x-p}$ where weights are given by the product of the group’s population share and its post-payment income share, α_j_.

6 $$H=\sum \limits_j{\alpha}_j{G}_j^{x-p}$$

*H* is non-negative since the Gini coefficient for each group of prepayment is non-negative. Thus, horizontal inequity will always make a post-payment distribution of income more unequal than it would have been in its absence. The reranking of households that occurs in the move from prepayment to post-payment income distributions is captured with *R*. The latter is measured as the difference between the Gini index for post-payment income *G*^*X-P*^ and the concentration index for post-payment income *C*^*x-p*^.

7 $$R={G}^{x-p}-{C}^{x-p}$$

When *R* is zero, there is no reranking in the transition from prepayment to post-payment periods causing the two curves to coincide. Therefore, *R* cannot be negative because the concentration curve of post-payment income cannot lie below the Lorenz curve of post-payment income.

Aronson et al., [1] decomposition shows that the total contribution of the health care payment system to income inequality can be decomposed into vertical equity, which represents the degree of progressivity of the health care financing system. A progressive health care financing system exerts an equalizing effect on post-payment income distribution. Horizontal inequity resulting from the health care financing system is estimated as the level of inequality in the post-payment income. The weighted sum of the within-group Gini coefficient gives the level of horizontal inequality *H* in the post-payment distribution. The last component is reranking among households as they move from the prepayment income distribution to post-payment income distribution.

### Data sources and variable definition

The data used for this study is drawn from the Second Cameroon Household Survey (ECAM IV) conducted in 2014 by the National Institute of Statistics. ECAM IV is a multipurpose household survey covering all ten regions of Cameroon and urban and rural areas using a sample of 12,847 households distributed in 1024 clusters or survey areas in 12 regions covering the national territory. The survey was designed to measure socio-economic factors relevant to the standards of living. The survey includes information on household characteristics, various sources of income, household expenditures on goods and services, including health and education. Furthermore, detailed information was collected on expenses on health care expenses.

Household prepayment income is measured by total household consumption, gross of out-of-pocket payment for health services. Household post-payment income so defined net of out-of-pocket payments. Prepayment and post-payment incomes are both defined to be gross food consumption on a per capita basis. The decomposition analysis was done at the level of the household. The method calculates the distribution of income before and after payments to provide a better insight into the impact of health care payments on the income distribution of households in Cameroon. In the calculations, households were divided into bands of prepayment income in which they were considered equals. Altogether, a total of sixteen bands were generated using multiples of the poverty line established in 2014.

## Results

Out-of-pocket payments for health services and the extent to which such payments affect the distribution of post-payment income determine the fairness of the health care system. For example, although the treatment of an illness episode can help restore an individual’s previous health status, if the payments compromise the household’s ability to afford other services, especially food, the situation becomes a great concern.

According to estimates from the Cameroon National Institute of Statistics, each Cameroonian household spent an annual average of 59,163 FCFA on health care, about 9860 CFA francs per person in a family of six (INS 2015). Health care expenditures rose by 6.8% between 2007 and 2014, imposing a huge financial health care access cost to households. In addition, the rise in health care payments can aggravate existing inequalities in the distribution of income. Household out-of-pocket payments on health care in Cameroon represent 70.6% of the total financing of the health sector in 2014.

The Gini coefficient for household consumption expenditure increased by 13% from 0.39 in 2007 to 0.44 in 2014 (INS 2015). Also, the decline in absolute poverty from 39.9% in 2007 to 37.5% in 2014 was not accompanied by any reduction in income inequalities between these two time periods. Therefore, the high Gini coefficient shows the limitation of the poverty reduction strategies implemented to reduce inequalities in the distribution of incomes in Cameroon.^1^

The analysis of the progressivity and redistributive effects of health care payments in Cameroon is based on data from the fourth Cameroon Household Survey. The sharing unit, as well as the unit of analysis, is the household. The sample used for the analysis has been weighted using sampling weights. On average, out-of-pocket payments absorbed about 7.77% of total household expenditures. Higher-income groups in Cameroon use health services in greater quantities so that higher income is associated with greater utilization of services and greater out-of-pocket payments on health care. It is also necessary to highlight that health care payments reflect illness reporting, which is biased in favor of richer households.

Table 3 shows the values of income *X*, out-of-pocket payments *(T)*, the income share of out-of-pocket payments g, the Gini coefficient for prepayment income *G*_*X*_, the concentration index for out-of-pocket payments *C*_*T*_, the concentration index for post-payment income to prepayment income *C*_*X-T*_, and the Kakwani index of progressivity of out-of-pocket payments on prepayment income ${\pi}_T^K$.

**Table 3:** Progressivity Indexes for Out-of-pocket payments for health care, 2014

Measure	Formula	Value
Gini for prepayment income	G _X_	0.45744
Gini for post-payment income	G _X-T_	0.45600
Redistributive effect	RE = Gx –G _X-T_	0.00144
Mean out-of-pocket payment (in FCFA)	T	28,620
Mean pre-payment income (in FCFA)	X	392,440
Mean fraction of prepayment income spent on health care	g = T/X	7.3%
Concentration index post-payment income	C _X-T_	0.45530
Concentration index payments (assuming within-group equality)	C _T_	0.48715
Kakwani index (assuming within-group equality)	${\pi}_T^K={C}_T-{G}_X$	0.02971

Computed by the author from ECAM IV data files using STATA 11.0

Households were then regrouped into these groups of prepayment equals. The concentration index for post-payment income (*C*_*X-T*_ = 0.4553) was then computed from groups of prepayment income. Finally, the Kakwani index (${\uppi}_{\mathrm{T}}^{\mathrm{K}}$ *= 0.02971*) was calculated as the difference between the payment concentration index *C*_*T*_ and the Gini coefficient *G*_*X*_.

Table 4 shows the Reynolds-Smolensky index of redistributive effects of out-of-pocket payment to pre-payment income (${\pi}_T^{RS}$ *= 0.03204*), the vertical redistributive effect (*V = 0.00234*), the horizontal equity (*H = 0.0002*), and re-ranking (*R = 0.0007*).

**Table 4:** Composition of Redistributive effect of out-of-pocket health care payments in Cameroon, 2014

Measure	Formula	
Redistributive effect	RE = Gx –G _X-T_	0.00144
Reynolds Smolensky (RS) Index	${\pi}_T^{RS}=\frac{1}{1-g}{\pi}_T^K$	0.03204
Vertical Redistribution Effect	$V=\frac{g}{1-g}{\pi}_T^K$	0.00234
Horizontal equity (computed as Residual)	*H* = *V* − *R* − *RE*	0.0002
Re-ranking	R = G_X-T_ – C_X-T_	0.0007
Sum of H and R	*H* + *R*	0.0009

Computed by the author from ECAM IV data files using STATA 11.0

The values of *G*_*X-T*_ and *C*_*X-T*_ are almost the same implying an insignificant reranking. The horizontal equity (*H = 0.0002*) offsets the disequalising effect of vertical income redistribution *(V = 0.00234)*. The choice of bandwidth has an effect on the computed values of *H* and *R*. As the bandwidth is widened, horizontal inequity falls, and reranking rises. Consequently, it becomes necessary to emphasize the sum of *H* and *R* rather than on their respective individual values.

The calculations in Tables 3 and 4 indicate that out-of-pocket payments for health care in Cameroon in 2014 were estimated at 7.3% of the total household expenditures and represent a significant share of household prepayment income. The estimates also show that the redistributive effect is positive, implying that health care payments are slightly progressive and will weakly enhance equity. However, the total effect on the distribution of disposable income is weak and almost negligible. Although out-of-pocket payments for health care in Cameroon represent a significant share of household prepayment income, the results of the study show that total health care payments were slightly progressive and have little effect on the distribution of post-payment income in Cameroon.

The estimated value of the Kakwani (${\uppi}_{\mathrm{T}}^{\mathrm{K}}$ = 0.0297) is positive, indicating a progressive payment structure. Also, the Reynolds-Smolensky index *(*${\uppi}_{\mathrm{T}}^{\mathrm{RS}}$*)* is positive, implying that the concentration curve for post-payment income *(Lpcexp)* lies above the Lorenz curve for prepayment income *(Lpre)* in Fig. 1, indicating in effect that out-of-pocket payments for health services do not result in inequality in the distribution of post-payment income. However, the low values of the Kakwani index *(*${\uppi}_{\mathrm{T}}^{\mathrm{K}}$*)* and Reynolds-Smolensky index *(*${\uppi}_{\mathrm{T}}^{\mathrm{RS}}$*)* suggest that post-payment reranking is low. Figure 1 shows the concentration curve of health care payments and the Lorenz curve of prepayment income variables, which are both plotted against the cumulative proportion of the sample ranked by income on the horizontal axis.

As a percentage of the RE, the vertical redistributive effect *V* is approximately 14%, indicating that in the absence of horizontal differences and reranking, the pro-poor income redistribution associated with out-of-pocket payments would have been only 14% of its actual value. The reason is that a pro-poor policy targets poor people, and there are more poor people receiving a benefit than non-poor [6]. In the case of health care, pro-poor policies increase access to free and affordable health care. Although the two indices are not equal, they are quite close, indicating low reranking resulting from payments. The estimated horizontal differences arise probably because of different levels of utilization at a given prepayment income level, partly attributed to differences in illness severity or because of different prices paid per unit of service. The latter may reflect differences in quality, especially in the for-profit and not-for-profit private sectors.

## Discussion

Evidence from the research suggests that health care financing in Cameroon through households’ out-of-pocket payments contributes to a slight reduction in income inequality. The positive redistributive effect associated with out-of-pocket payments is consistent with findings reported in Sierra Leone (Fabricant et al. [8]), in Ghana, Burkina Faso (Makinen et al.[14]), in South Africa, and Tanzania [17], While these studies examine overall sources of health care financing, this research focuses only on households’ out-of-pocket payments.

Like in Ataguba and McIntyre’s [5] study on South Africa, Mills et al. [17] in Ghana, South Africa, and Tanzania, Mondaca and Chi [19] in Chile, the progressive financing system of health care through out-of-pocket payments in Cameroon is pro-rich in terms of health care benefits. The rich consume more health care in Cameroon than the poor and thus incur more out-of-pocket costs. The low per capita income for a large segment of the Cameroon population and the high incidence of poverty (37.5% in 2014) expose poorer households to high out-of-pocket expenses and limit access to health services. Health care financing through direct taxes may result in a reduction in income inequality. Furthermore, allocating more public expenditure to improve health infrastructure and services could expand access to health care and reduce inequity.

However, insufficient government funding of health care can result in escalating health care costs for low-income households. The allocation of scarce public resources to the health sector favors high-level curative services at the expense of intermediate and peripheral levels and can potentially increase households’ out-of-pocket for critical health services. This also tends to escalate inequity in access to health care services and the distribution of post-payment incomes.

Although health care spending in Cameroon is progressive, it is not pro-poor and tends to benefit more affluent people through higher utilization of health care services [34]. It is worthwhile to underscore that in the 2014 ECAM 4 households survey, health care payments reflect illness reporting, predominantly by richer households. Thus, the progressivity of out-of-pocket payments is not coincidental, since more affluent households use more care and report illness than poorer households.

In the current study, the progressive vertical effect dominates both the horizontal effect and reranking. Out-of-pocket payment in the absence of a well-developed and expanded private insurance can lead to catastrophic financial outcomes and impoverishment. Increases in government resources to health care and equity in the distribution of health infrastructure across geographical areas and facilities will expand access to health services to all populations and reduce out-of-pocket payments, especially for lower-income groups.

## Conclusion

This study has found that the redistributive effect of out-of-pocket household payments for health services in Cameroon is positive, indicating that health care payments are to an extent progressive. Second, according to the findings, the reranking of households is quite negligible, implying that very few household members change position in the distribution of income after paying for health services. The third important finding is that there exists some amount of horizontal inequity among members of the same income bands, and this can be explained by differences in the levels of utilization at a given prepayment income level. The differences in the level of utilization are attributed to differences in illness severity and perception, the level of prices paid per unit, and the economic position of the health care users.

The findings from the study should be interpreted with care as the inequality in access and payments for health care in Cameroon are proportional to the ability to pay for health services. The high utilization of health services by people with better economic positions translates into an expenditure pattern in which payments increase with incomes, with the richest spending close to twelve times on health care than the poorest quintile [34]. The low utilization of health services by lower-income groups despite higher health care needs is due to the lack of access to health services and the inability to afford the high cost of services.

The study has shown that out-of-pocket payments on health care in Cameroon affect income inequity within and across income groups and thus are relevant for policy discussions on health care financing options. Furthermore, the progressivity of out-of-pocket payments indicates the existence of considerable barriers to health care access to low-income groups. Thus, the financial barriers for low-income groups can be dismantled through more government allocations to the health sector to expand access to quality and affordable care.

Further studies of the redistributive effects of health care financing in Cameroon should include other sources of health care financing such as direct taxes, indirect taxes, in addition to out-of-pocket payments. It is equally important to compare the relative income redistributive effects of public versus private health care markets, given that these markets charge different prices for services and provide services that differ in quality. Finally, further studies should explore equity in access and utilization of health care services.

## Data Availability

Household survey data was obtained from the Cameroon National Institute of Statistics.

## Footnotes

1 Poverty incidence has reduced, but differences in the distribution of household consumption across the country show severe inequalities.

## References

[1] Aronson JR, Johnson P, Lambert PJ (1994). Redistributive effect and unequal income tax treatment. Econ J.

[2] Asante A, Price J, Hayen A, Jan S, Wiseman V (2016). Equity in health care financing in low- and middle-income countries: a systematic review of evidence from studies using benefit and financing incidence analyses. PLoS One.

[3] Asante A, Wasike WSK, Ataguba JE (2020). Health financing in sub-Saharan Africa: from analytical frameworks to empirical evaluation. Appl Health Econ Health Policy.

[4] Ataguba JE, Ichoku HE, Nwosu CO, Akazili J. An alternative approach to decomposing the redistributive effect of health financing between and within groups using the Gini Index: the case of out-of-pocket payments in Nigeria. J Appl Health Econ Health Policy. 2019. 10.1007/s40258-019-00520-4. (Epub 18 Oct 2019). 10.1007/s40258-019-00520-4 PMC7716861 31628664

[5] Ataguba JE, McIntyre DI. Paying for and receiving benefits from health services in South Africa: is the health system equitable? Health Policy Plann. 2012; 10.1093/heapol/czs005 Download as RIS. 10.1093/heapol/czs005 22388498

[6] Curran Z, De Renzio P. What do we mean by “pro-poor policies” and “pro-poor policy processes”? London: ODI; 2006.

[7] Deaton A, Muellbauer, J. Economics and Consumer Behaviour. Cambridge University Press; 1980.

[8] Fabricant SJ, Kamara CW, Mills A. Why the poor pay more: Household curative expenditures in rural Sierra Leone. International Journal of Health Planning and Management, 1999;14:179-99. 10.1002/(SICI)1099-1751(199907/09)14:3<179::AID-HPM548>3.0.CO;2-N 10623188

[9] Gonzalez Pier E, Parker S (1999). Equity in the finance and delivery of health care: results from Mexico.

[10] Holst J (2012). Direktzahlungen in der Krankenversorgung in Entwicklungs- und Schwellenländern: Ein Reforminstrument mit überwiegend negativen Wirkungen.

[11] Institut National de Statistiques (INS). Quatrième Enquête camerounaise auprès des menages (ECAM 4) : premiers résultats; 2015.

[12] Kakwani K (1977). Measurement of tax progressivity: an international comparison. Econ J.

[13] Litvack IJ, Bodart C. User Fees Plus Quality Equals Improved Access to Health Care: Results of a Field Experiment in Cameroon Social Science and Medicine. 1993;37:369-383. 10.1016/0277-9536(93)90267-8 8356485

[14] Makinen M, Waters H, Rauch M, Almagambetova N, Bitran R, Gilson L, et al. Inequalities in health care use and expenditures; empirical data from eight developing countries and countries in transition. Bulletin of the World Health Organization. 2000;78:55-65. PMC2560608 10686733

[15] McIntyre D, Gilson L, Mutyambizi V (2005). Promoting equitable health care financing in the African context: current challenges and future prospects.

[16] McIntyre D, Garshong B, Mtei G, Meheus F, Thiede M, Akazili J, Ally M, Aikins M, Mulligan J-A, Goudge J (2008). Beyond fragmentation and towards universal coverage: insights from Ghana, South Africa and the United Republic of Tanzania. Bull World Health Org.

[17] Mills A, Ataguba JE, Akazili J, Borghi J, Garshong B, Makawia S, Mtei M, Harris B, Macha J, Meheus F, Mclntyre D (2012). Equity in financing and use of health care in Ghana, South Africa, and Tanzania: implications for paths to universal coverage. Lancet.

[18] Ministry of Public Health (MOH), Health Sector Strategy (HSS), 2016-2027, Yaounde, Cameroon, 2016.

[19] Mondaca ALN, Chi C (2017). Equity in out-of-pocket payment in Chile. Rev Saúde Pública.

[20] Munge K, Briggs AH (2014). The progressivity of healthcare financing in Kenya. Health Policy Plan.

[21] Musgrave RA, Thin T (1948). 1948. Income tax progression, 1929-48. J Polit Econ.

[22] Ntembe NA. User Charges and Health Care Provider Choice in Cameroon. International Review of Business Research Papers. 2009;5 (6):33-49.

[23] Pannarunothai S, Mills A (1997). The poor pay more: health-related inequality in Thailand. Soc Sci Med.

[24] Van Doorslaer E, Wagstaff A (1999). The redistributive effect of health care financing in twelve OECD countries. J Health Econ.

[25] Wagstaff A, Flores G, Smitz MF, Hsu J, Chepynoga K, Eozenou P (2018). Progress on impoverishing health spending in 122 countries: a retrospective observational study. Lancet Glob Heal.

[26] Wagstaff A (2000). Measuring equity in health care financing: reflections on and alternatives to WHO's fairness of financing index.

[27] Wagstaff A, van Doorslaer E. 'Catastrophe and impoverishment in paying for health care: with applications to Vietnam 1993–1998' Health Economics. 2003;12(11):921-33. 10.1002/hec.776. 10.1002/hec.776 14601155

[28] Wagstaff A, van Doorslaer E (1997). Progressivity, horizontal equity and reranking in health care finance: a decomposition analysis for the Netherlands. J Health Econ.

[29] Wagstaff A, van Doorslaer E. Equity in the Finance of Health Care: Some Further International Comparisons. J Health Econ. 1999;18:263-90. 10.1016/s0167-6296(98)00044-7 10537896

[30] Wagstaff A, van Doorslaer E (1993). Equity in the finance and delivery of health care: an international perspective.

[31] Wagstaff A, Doorslaer E, Paci P. Equity in the Finance and Delivery of Health Care: Some Tentative Crosscountry Comparisons, Oxford Review of Economic Policy, Oxford University Press. Spring. 1989;5(1),:89-112.

[32] Wagstaff A, van Doorslaer E (2001). Pay for health care: quantifying fairness, catastrophic and impoverishment, with applications to Vietnam 1993-98.

[33] Wagstaff A. Econometric studies in health economics: A survey of the British literature. Adam Wagstaff · J Health Econ. 1989;8(1):1-51. 10.1016/0167-6296(89)90008-8 10303557

[34] World Bank (2018). Cameroon-public expenditure review: aligning public expenditures with the goals of vision 2035 (English).

[35] Wagstaff A. Poverty, and Health Sector Inequalities. Bulletin of the World Health Organization 2002;80;97-105. PMC2567730 11953787

